# Foodborne Parasites Dominate Current Parasitic Infections in Hunan Province, China

**DOI:** 10.3389/fcimb.2021.774980

**Published:** 2021-10-14

**Authors:** Xiaohua Liu, Mengqi Wu, Yuan Liu, Jing Li, Dongqian Yang, Liping Jiang

**Affiliations:** ^1^ Department of Parasitology, Xiangya School of Medicine, Central South University, Changsha, China; ^2^ China-Africa Research Center of Infectious Diseases, Xiangya School of Medicine, Central South University, Changsha, China

**Keywords:** parasitic infection, foodborne parasites, prevalence, risk factors, Hunan Province

## Abstract

Great progress has been made in the prevention and treatment of human parasitic diseases in China over the past six decades, but parasitic diseases are still one of the most serious public health problems in the world. The specific prevalence of parasitic diseases varies in different provinces due to their geographical environment and the dietary habits of people. In this study, a total of 4,428 patients suspected to have parasitic infection by clinicians or themselves from January 1, 2016, to December 31, 2020 were recommended to our laboratory for further testing. In total, 5,246 samples including fecal, blood, and other body fluids were detected by etiological and immunological methods. Approximately 15.20% (673/4,428) of all suspected patients were infected by at least one species of parasite, and the overall positive rate of suspected patients from Hunan Province was 15.10% (594/3,933). A total of 18 species of parasites, namely, nematodes (4 species), trematodes (5 species), cestodes (4 species), protozoa (2 species), and medical arthropods (3 species), and 3 of them were imported parasites outside of Hunan Province. There are 9 species of foodborne parasites, accounting for 89.92% (464/516) of patients infected by one species of parasite. Common parasites in Hunan Province include plerocercoid, *Paragonimus westermani*, *Clonorchis sinensis*, cysticercus, *Toxoplasma gondii*, and *Schistosoma japonicum*. In this study, we found that the incidence of soilborne nematode infections has decreased significantly. However, foodborne parasites gradually become the main parasitic infections as well as multiple infections are becoming more common. Therefore, we should not only continue the prevention and control of soil-derived nematodes but also focus on the prevention and control of foodborne parasites in the future.

## 1 Introduction

Parasites can cause a wide variety of serious economic and public health problems. The Ministry of Health of the People’s Republic of China conducted three nationwide surveys on the prevalence of human parasitic diseases in 1990, during 2001–2004, and 2014–2016, respectively, which showed that the positive rate of soil-transmitted nematodes, such as hookworm, *Ascaris lumbricoides* (*A*. *lumbricoides*), and *Trichuris trichiura* (*T*. *trichiura*), was significantly reduced, but the positive rate of foodborne parasites was significantly increased in some provinces (Yu et al., 1994; Xu et al., 1995; Coordinating Office of the National Survey on the Important Human Parasitic, 2005; Zhu et al., 2015). In 2006, soil-transmitted nematodiasis was included in the national infectious diseases and pathogenic media monitoring system by the Chinese Center for Disease Control and Prevention, and 22 monitoring spots were established nationwide, showing a declining trend of the human positive rate of intestinal nematodes from 2006 to 2013 (Chen and Zang, 2015). Over the years, the work of health education actively carried out by the Ministry of Health and periodic drug treatment for patients with parasitic infections prevent reinfection and reduce morbidity greatly (Zhao et al., 2012; McManus et al., 2014; Rivero et al., 2017; Guang-Han et al., 2018; Laoraksawong et al., 2018). Health education in primary schools showed a significant efficacy in preventing intestinal parasitic infections (Bieri et al., 2013a; Bieri et al., 2013b; Bieri et al., 2014; Cisse, 2019). The rapid economic growth over the past six decades witnessed the increased allocation of resources toward the control of parasitic diseases. Consequently, the status of parasitic diseases in China has improved significantly (De-Jian et al., 2013; Wang et al., 2016).

Foodborne parasites, most of which are zoonotic, can be transmitted by ingesting contaminated food and water. According to the life cycle and transmitted mode of parasites, foodborne parasites mainly include *Trichinella*, protozoa [e.g., *Toxoplasma gondii* (*T. gondii*), *Giardia lamblia*, and *Cryptosporidium*], tapeworms (e.g., *Taenia solium* and *Spirometra mansoni*), and trematodes [e.g., *Paragonimus westermani* (*P. westermani*), *Clonorchis sinensis* (*C. sinensis*), *Schistosoma*, and *Fasciolopsis buski* (*F. buski*)] (Robertson et al., 2014; Pozio, 2020). In 2006, the World Health Organization (WHO) established the Foodborne Disease Burden Epidemiology Reference Group (FERG), and in 2013, they summarized the burden of foodborne diseases (FBDs) and the important results available to date (Torgerson et al., 2014). The Global Burden of Disease Study published the global burden of foodborne trematodiasis and its sequelae in 2012 (Furst et al., 2012), showing that about 56.2 million people were infected with foodborne trematodes.

Great progress has been made in the prevention and treatment of human parasitic diseases in China over the past six decades. On June 30, 2021, the World Health Organization announced that malaria has been eliminated in China, but parasitic diseases are still one of the most serious public health problems in the world. China is still affected by parasitic infections, including leishmaniasis, schistosomiasis, toxoplasmosis, and other foodborne nematodiasis (Li et al., 2010; Wang et al., 2016). With increased income standards of living and consumption of exotic foods, foodborne parasitic infection has become one of the main factors that impact upon national food safety and public health (Li et al., 2010). The main foodborne parasitic diseases in China include paragonimiasis, clonorchiasis, toxoplasmosis, angiostrongyliasis, echinococcosis, trichinellosis, and cysticercosis (Torgerson et al., 2014). The overall prevalence of *T. gondii* infections in food animals was significantly higher than that in humans (Dong et al., 2018). In Hunan Province, because of the dietary habits of local residents, such as eating raw snake galls and undercooked fish and crabs, the status of some foodborne parasitic infections is serious. Due to the strengthening of international cooperation in globalization and the prosperity of tourism, imported cases are also increasing (Song et al., 2018).

In 2015, the investigation on the prevalence of major human parasitic diseases in Hunan Province showed that soilborne nematode infection accounted for 80.35% of the intestinal parasite infections (Zhuo et al., 2017), which suggested that we still need to strengthen the prevention and control of soilborne nematodes. In Hunan Province, the parasite epidemic situation in the recent 5 years is unclear. Through retrospective analysis of the examination of suspected cases of parasitic diseases in our laboratory from January 1, 2016, to December 31, 2020, our study aims to understand the current situation and prevalence of parasitic diseases and infection in Hunan Province and provide scientific data and basis for the prevention and treatment of parasitic diseases in the future.

## 2 Materials and Methods

### 2.1 Participants and Samples

A total of 5,246 samples including fecal, blood, and other body fluids of 4,428 inpatients and outpatients were detected in the Parasitological Laboratory of XiangYa School of Medicine from January 1, 2016, to December 31, 2020, and there were 3,933 patients from Hunan Province. The total number of cases in 2020 is relatively small as no samples were tested from February to April due to coronavirus disease 2019 (COVID-19). Patients suspected to have a parasitic infection by their clinician or themselves were recommended to our laboratory for further testing. Therefore, the positive rate of these patients may be higher than that of stratified cluster random sampling method. We recorded the basic information of the patients such as sex, age, region, and medical history while we received specimens. Some patients had different specimens examined at the same time. If multiple test results of the same patient were different, positive results were taken and the number of positive patients was counted.

### 2.2 Pathogen Detection

Pathogen detection is the direct evidence of parasitic infections. The detection methods include saline direct smear, iodine staining smear method, saturated saline floatation, Kato’s thick smear, blood smear, bone marrow smear, and so on. We observe whether there are worms, eggs, trophozoites, or cysts in specimens through a microscope.

#### 2.2.1 Direct Saline Smear Method

A drop of normal saline or iodine solution is dripped on a clean slide and a small amount of feces is picked up with a bamboo stick, then it is smeared evenly in the normal saline and covered with the cover glass. The slide is placed under a microscope and observed at low and high magnifications. The eggs or trophozoites are identified according to their size, shape, color, and motility characteristics.

#### 2.2.2 Iodine Staining Smear Method

The iodine staining smear method is mainly used to examine protozoa cysts. A drop of iodine is dripped on a clean slide and a small amount of feces is picked up with a bamboo stick, then it is smeared evenly in the iodine and covered with the cover glass. Cysts are stained yellow or light brownish yellow, glycogen bubbles are brownish red, while the walls, nucleoli, and chromatoid bodies are not stained.

#### 2.2.3 Saturated Saline Floatation

The saturated saline floatation is suitable for the examination of eggs with small specific gravity, such as hookworm eggs and tapeworm eggs. Feces of one soybean volume is taken with a bamboo stick into the floating bottle and a small amount of saturated saline is added, and then it is mixed evenly. Next, saturated saline is added until it rose to the top. Large impurities on the liquid surface are removed, and finally saturated saline is slowly added until the liquid becomes slightly higher than the bottle mouth, but does not overflow. The bottle is covered with a glass slide to avoid bubbles. After 15 min, the slide is lifted, turned over quickly, covered with the slide, and then observed under a microscope.

#### 2.2.4 Kato’s Thick Smear

The detection rate of Kato’s thick smear method is more than 20 times than that of the direct smear method. Fifty to 60 g feces is put on a slide, covered with cellophane soaked with glycerin-malachite green solution, and pressed lightly to make the feces spread about 20 mm 25 mm. Next, it is put in a temperature box at 30°C–36°C for 30 min or 25°C for 1 h. Microscopic examination can be conducted after the feces film is transparent.

#### 2.2.5 Blood Smear and Bone Marrow Smear

Blood smears, with thick and thin blood films on the same slide, are routinely used to diagnose plasmodium. Bone marrow smear is mainly used to examine the amastigote of *Leishmania donovani (L. donovani)*. The thin blood film is made by placing a small drop of blood at the junction of one-third and two-thirds of the slide with the other slide in front of the blood drop and the angle between the two slides being 30°–45° and by pushing the slide back at a constant speed to make the thin blood film. The ideal thin blood smear should be a uniformly distributed layer of blood cells with no space between them, and the ends of the blood membrane should be broom-like. The thick blood film is made by placing a drop of blood on the right one-third of the slide and rotating the corner of the slide from inside to outside to make it into a thick blood film with a diameter of 0.8–1 cm and uniform thickness. After the blood smear is naturally dried, two to three drops of distilled water need to be added to the thick blood film to cause its hemolysis. When the blood smear turns gray, the water is poured away, and the blood smear is left to be dried. The thin and thick blood films are fixed with methanol, then stained with Giemsa solution for 25–30 min, and finally washed with water or buffer solution, dried, and examined under a microscope.

### 2.3 Determination of Antibodies Against Parasites

About 5 ml of venous blood is collected from each patient by a health professional. The supernatants are separated from the whole blood or other body fluids by centrifuging at 5,000 rpm for 5 min and then stored at 4°C for further testing.

#### 2.3.1 Indirect Hemagglutination Assay

##### 2.3.1.1 IHA Kit for Detection of Schistosomiasis japonicum

Antibodies against *S. japonicum* are mainly detected by indirect hemagglutination assay (IHA), using the IHA kits (Anji Medical Technology Co., Ltd., Anhui Province, China). The *S. japonicum* soluble egg antigen (SEA) is adsorbed on the red blood cell carrier to make it become sensitized red blood cell. When the sensitized red blood cell meets the antibody in the serum of the patient, the antigen adsorbed on the red blood cell surface and the specific antibody are combined under appropriate conditions to form the visible red blood cell agglutination phenomenon, which is a positive reaction. The test was performed following the instructions of the manufacturer.

##### 2.3.1.2 IHA Kit for Detection of Toxoplasma gondii

IHA kits are provided by Lanzhou Veterinary Research Institute, Chinese Academy of Agricultural Sciences, Lanzhou, Gansu Province, China. The test is performed following the instructions of the manufacturer. This kit is a lyophilized antigen for IHA of *T. gondii* with a positive serum titer of no less than 1:1,024. Ten samples can be detected per milliliter of antigen for qualitative examination, while only five samples can be detected per milliliter of antigen for quantitative examination.

#### 2.3.2 Diagnostic Kit for Antibody to *Schistosoma* Egg

The colloidal gold method for the detection of *S. japonicum* egg antibody is used as an auxiliary examination by the DIGFA Kits (Xunchao Biotech Co., Ltd., Yueyang, China). Purified *S. japonicum* egg-specific protein antigen is used to spot on the filter membrane, and colloidal gold is used to label staphylococcal protein A (SPA). Based on the principle of colloidal gold mononitration speck method, when the tested sample is filtered from the membrane, the *S. japonicum* egg antibody fixed on the membrane and the specific antibody in the test sample form an antigen–antibody complex, which forms visible red spots with the SPA-labeled colloidal gold. The assay is performed following the instructions of the manufacturer.

#### 2.3.3 Enzyme-Linked Immunosorbent Assay

The enzyme-linked immunosorbent assay (ELISA) kits (Shenzhen Combined Biotech Co., Ltd., Shenzhen, China) are classified as types A, B, and C. We use indirect ELISA detection, according to the instructions of the manufacturer (http://www.biacbd.com/). In short, firstly, properly diluted specimens are added into micropores of the envelope antigen; after incubation and washing, the enzyme-labeled second antibody is added to produce a compound of antigen, antibody to be tested, and the enzyme-labeled second antibody; subsequent to washing, the enzyme conjugate not adsorbed is removed, which is followed by the addition of a substrate and a color developing agent. In this way, absorbance of the inspection hole and the control hole is tested so as to determine whether specific antibodies exist in the specimen. Key components of ELISA are solid phase vectors and enzyme conjugates that adsorb antigens or antibodies. Moreover, their behavior can directly influence detection results.

### 2.4 Statistical Analysis

The cases were categorized according to their sex, age, region, clinic time, and so on. Comparisons of the effects of sex, age, region and clinic time on prevalence of parasitic infections were performed by Chi-square tests in contingency tables using GraphPad Prism version 5.0 (GraphPad Software, San Diego, CA, USA). When *P*-value <0.05, the difference was considered significant.

### 2.5 Map

The map of Hunan Province is downloaded from the standard map service website (http://bzdt.ch.mnr.gov.cn/).

### 2.6 Ethical Review

The study involving human participants were reviewed and approved by the Ethics Committee of the School of Basic Medical Science, Central South University, Changsha, China (protocol codes syxk2011-0001 and 2017-S088, dates of approval March 3, 2015, and March 8, 2017, respectively). Written informed consent to participate in this study was provided by the legal guardian/next of kin of the participants. For data that we used in this study, personal information had been removed and no specific individuals could be identified. No potentially identifiable human images or data are presented in this study. The raw data supporting the conclusions of this article will be made available by the authors, without undue reservation.

## 3 Results

### 3.1 The Results of Specimen Examination

**Figure 1:**
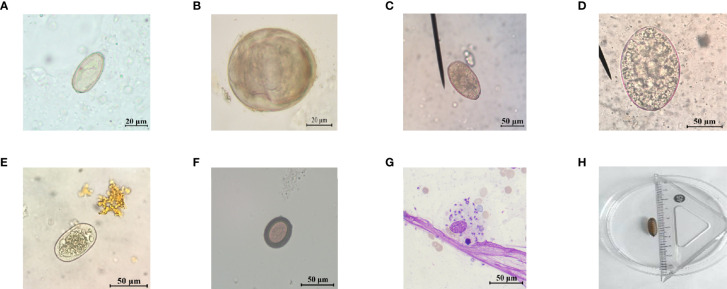
Representative images of parasite eggs, amastigotes, and larva for etiological examination. **(A)** *C. sinensis* egg; **(B)** *S. japonicum* egg; **(C)** *P. westermani* egg; **(D)** *F. buski* egg; **(E)** hookworm egg; **(F)** *Taenia* egg; **(G)** *L. donovani* bodies; and **(H)** maggot.

In this study, a total of 4,428 suspected patients were received, including 2,399 patients from Hunan Province, 296 patients from other provinces, and 199 patients whose hometown was unknown. At least one species of parasites was identified in 15.20% (673/4,428) of patients, and the overall positive rate of patients from Hunan Province was 15.10% (594/3,933), while the positive rate of patients from other provinces was 17.23% (51/296) and that of patients whose hometown was unknown was 14.07% (28/199). Etiological examination was used for feces and smears, to observe whether there were worms, eggs, or trophozoite of protozoa in specimens through a microscope (**Figure 1**), and 28 positive cases were confirmed including hookworm (6 cases), *F. buski* (6 cases), *Enterobius vermicularis* (1 case), *A*. *lumbricoides* (1 case), *C. sinensis* (5 cases), *S. japonicum* (2 cases), *P. westermani* (1 case), *Phthirus pubis* (1 case), maggot (2 cases), *Psychodidae* (1 case), *Taenia saginata* (*T. saginata*) (1 case), and *Leishmania* (2 cases), with a positive rate of 6.26%. As for blood or other body fluid samples, we mainly adopted immunological examination, and there was a relatively higher positive rate of 16.12% (**Table 1**).

**Table 1:** The results of all samples tested.

Types of samples	Method	No. of examined	No. of positive	Positive rate (%)
Feces or smears	Etiological test	447	28	6.26
Blood or other body fluid	Immunological test	4,801	774	16.12
Total		5,248	802	15.28

We found 18 species of parasites (**Table 2**), namely nematodes (4 species), trematodes (5 species), cestodes (4 species), protozoa (2 species), and arthropods (3 species). There were nine species of foodborne parasites, accounting for 89.92% (464/516) of detected single parasites, and only eight cases of soilborne nematodes were detected in this study. As shown in **Table 2**, trematodes and tapeworms predominated in infection, while nematodes were rarely found. The most common parasites included *P. westermani*, plerocercoid, cysticercus, *C. sinensis*, *T. gondii*, and *S. japonicum*, among which *P. westermani* and plerocercoid were more serious.

**Table 2:** The species of parasites detected and the positive rate.

Species of parasite	No. of positive patients (*n*)	Positive rate (%) (*n*/4,428)
Nematodes		
*Trichinella*	12	0.27
Hookworm	6	0.14
*Ascaris lumbricoides*	1	0.02
*Enterobius vermicularis*	1	0.02
Trematodes		
*Paragonimus westermani*	106	2.39
*Clonorchis sinensis*	70	1.58
*Schistosoma japonicum*	40	0.9
*Fasciolopsis buski*	6	0.14
*Schistosoma mansoni*	1	0.02
Cestodes		
Plerocercoid	116	2.62
Cysticercus	71	1.6
Hydatid cyst	20	0.45
*Taenia saginata*	1	0.02
Protozoa		
*Toxoplasma gondii*	60	1.36
*Leishmania donovani*	1	0.02
Medical arthropods		
Maggot	2	0.05
*Phthirus pubis*	1	0.02
Psychodidae	1	0.02
Multiple infection	157	3.55

We confirmed three imported cases outside of Hunan Province, one of which was Kala-azar, and the patient who had worked in the epidemic area of Gansu Province was a native of Daoxian County, Hunan Province. Another was *T. saginata*, and the patient who worked in Ethiopia was a native of Changsha. The last one was *Schistosomiasis mansoni* (*S. mansoni*) and the patient had worked in Africa.

### 3.2 Parasitic Infections Were More Common in Males Than in Females

**Figure 2:**
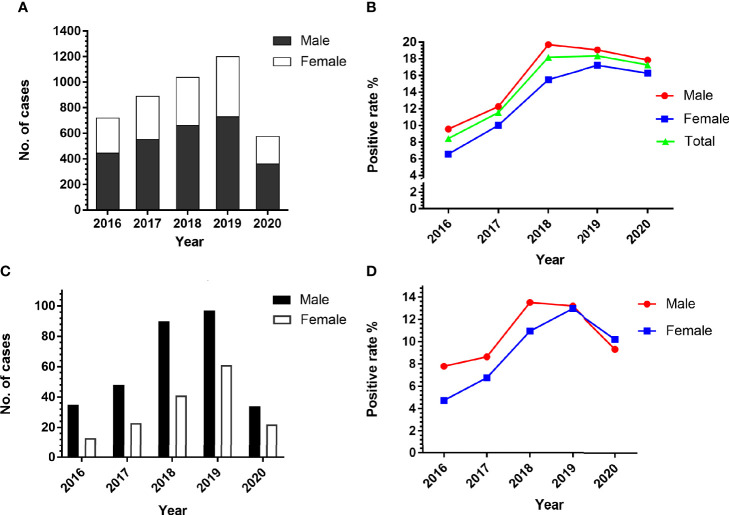
Comparison of the prevalence of parasites in patients according to sex. **(A)** The number of people suspected to have a parasitic infection. **(B)** The positive rate of male and female in the period of 2016 to 2020. **(C, D)** The number and positive rate of single foodborne parasites in patients according to sex.

Of the 4,428 patients, 447 of 2,756 males were positive for at least one parasite, with a positive rate of 16.22%. Similarly, 226 of 1,672 females were positive for at least one parasite, with a positive rate of 13.52%. In the period of 2016 to 2020, the number of males suspected to have a parasitic infection is more than that of females (**Figure 2A**). The total number of cases in 2020 is relatively small as no samples were tested from February to April due to the outbreak of COVID-19. The sex-specific prevalence showed a high incidence in males (9.58%–19.70%) compared with females (6.57%–17.23%) in all the years of this study (**Figure 2B**). In total, we detected 464 positive cases of single foodborne parasites, and the positive rate of males was significantly higher than that of females except in 2020 (**Figures 2C, D**). Although there was no significant difference in the prevalence of total parasites (*P* > 0.05) and foodborne parasites (*P* > 0.05) with reference to sex, there is a tendency for male infection to be higher and female infection to be lower than the overall positive rate.

### 3.3 The Species and Incidence of Parasitic Infections Vary With Age

**Figure 3:**
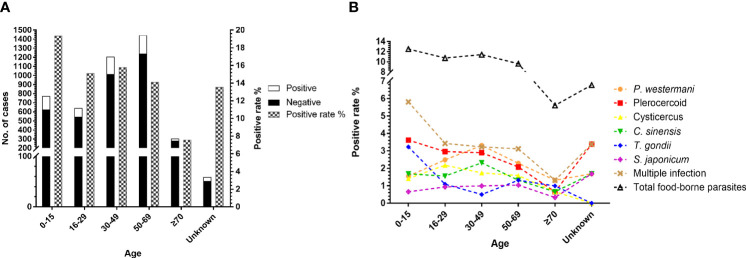
Comparison of the prevalence of parasites in patients according to age. **(A)** Number of cases received and the prevalence of total parasitic infection among age groups. **(B)** Comparison of the prevalence of the six most commonly detected parasites among age groups.

We categorized 4,428 cases into six different groups according to age. At least one parasite was detected in 150 of 774 patients less than 15 years old, 97 of 642 patients between 16 and 29 years old, 191 of 1,207 patients between 30 and 49 years old, 204 of 1,443 patients between 50 and 69 years old, 23 of 303 patients older than 70 years old, and 8 of 59 patients of unknown-age group (**Figure 3A**). There was a significant difference in the prevalence of total parasitic infection (χ^2^=25.71, df=4, *P*<0.0001) according to age. However, only plerocercoid (χ^2^=9.934, df=4, *P*<0.05), *T. gondii* (χ^2^=27.28, df=4, *P*<0.0001), and total foodborne parasites (χ^2^=13.39, df=4, *P*<0.01) showed a significant difference in the prevalence. As shown in **Figure 3B**, people in the 30–49 age group had the highest positive rate of *P. westermani* and *C. sinensis*, but the lowest positive rate of *T. gondii*. The positive rate of total foodborne parasites decreased with age. Although the positive rate of plerocercoid decreased with the increase of age, it still was the dominant parasite in all age groups. Children below 15 years old suffer more serious parasitic infection especially foodborne parasite infection and multiple infection.

### 3.4 The Prevalence of Parasites Was Influenced by Geography

**Figure 4:**
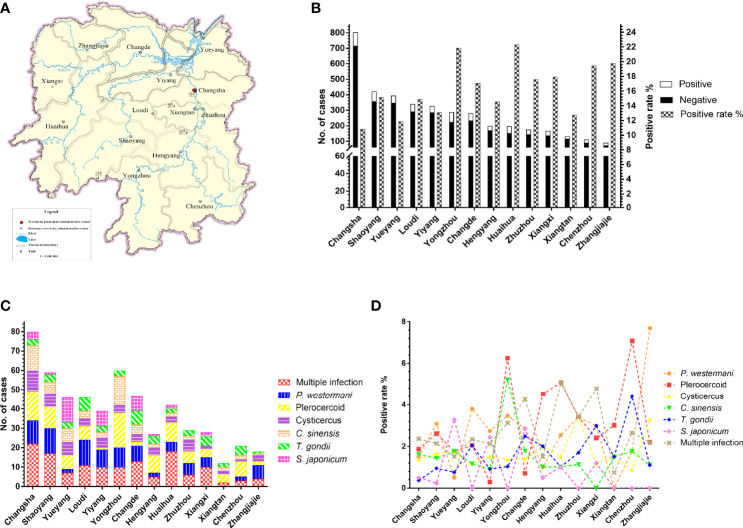
Prevalence of parasites in different cities in Hunan Province, China. **(A)** The map of Hunan Province, China. **(B)** The number of patients and total parasitic positive rate in 14 cities. **(C, D)** Distribution and prevalence of several common parasites in 14 cities.

The results of other provinces and unknown groups were excluded from the statistics examination. The 3,933 cases from Hunan Province were categorized into 14 groups according to cities (**Figure 4A**). The prevalence of parasites in nine cities was higher than that of total parasitic infection in Hunan Province (15.10%, 594/3,933) (**Figure 4B**). There were some differences in parasite species among the cities due to different topographical features. Although Changsha had the largest number of participants, its overall positive rate was the lowest among 14 cities in Hunan Province. The overall positive rates in Yongzhou and Huaihua were more than 20%. Apparently, the incidence of *S. japonicum* was high in Yueyang, Yiyang, and Changde (**Figures 4C, D**). Besides, *P. westermani* and plerocercoid were widely prevalent in the whole province, while the positive rate of *C. sinensis* was the highest in Yongzhou. Multiple infections were observed evidently in Huaihua (**Figure 4B**). There was a significant difference in the prevalence of total parasitic infection (χ^2^=40.48, df=13, *P*<0.0001) among 14 cities in Hunan Province. Besides, *P. westermani* (χ^2^=31.53, df=13, *P*<0.01), plerocercoid (χ^2^=45.57, df=13, *P*<0.0001), *C. sinensis* (χ^2^=28.92, df=13, *P*<0.01), *T. gondii* (χ^2^=24.89, df=13, *P*<0.05), and *S. japonicum* (χ^2^=54.55, df=13, *P*<0.0001) also showed significant difference in prevalence among the 14 cities.

### 3.5 Parasitic Positive Rates Were Varied by Seasonality

**Figure 5:**
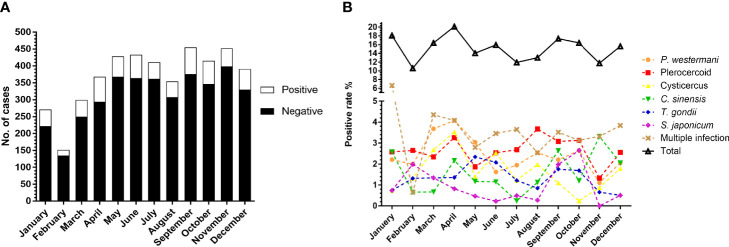
Comparison of the prevalence of parasites between months. **(A)** Number of cases received and results in different months. **(B)** Comparison of the prevalence of total parasites and the six most commonly detected parasites among the months.

To investigate whether parasitic infections were related to seasonality, we also analyzed the number of suspected patients and prevalence of parasitosis of different months. The number of case submittals was larger in spring and autumn. The highest and lowest prevalence occurred in April (20.11%, 74/368) and February (10.60%, 16/151), respectively (**Figure 5A**). There was a significant difference in the total parasitic infection (χ^2^=23.23, df=11, *P*<0.05) between months. As for the six common parasites, cysticercus, (χ^2^=22.63, df=11, *P*<0.05) *C. sinensis* (χ^2^=23.02, df=11, *P*<0.05), and *S. japonicum* (χ^2^=32.96, df=11, *P*<0.001) also showed significant difference among the months (**Figure 5B**).

## 4 Discussion

In this survey, people suspected to have a parasitic infection by clinicians or themselves were recommended to the Parasitological Laboratory, Department of Parasitology, Xiangya School of Medicine, Central South University, Changsha, Hunan, China, for further testing; therefore, the positive rate of parasitic infection of the survey may be higher than that of the stratified cluster random sampling method. In 2015, the investigation on the prevalence of major human parasitic diseases in Hunan Province showed that soilborne nematode infection accounted for 80.35% of intestinal parasite infections (Zhuo et al., 2017). However, we found that foodborne parasites account for a major portion of parasite infections, and only eight cases of soilborne nematodes were detected in this study, which suggests that over these years the work of controlling soilborne nematode infections had achieved great success. On the other hand, nematode infection is relatively easy to identify and can be easily diagnosed by clinicians, so it does not require further examination in our laboratory. That is why the number of stool samples accepted by our laboratory is relatively small, resulting in a relatively small proportion of nematodes detected. In previous studies, both the positive rates of overall parasites and soilborne nematodes of female patients were higher than those of male patients (Yu et al., 1994; Xu et al., 1995; Coordinating Office of the National Survey on the Important Human Parasitic, 2005; Zhu et al., 2015), which was opposite of our results. Although the number of soilborne nematodes we detected was too small to be analyzed effectively, the positive rate of males for both overall parasites and foodborne parasites was significantly higher than that of females.

In this survey, the main parasites detected were foodborne parasites such as plerocercoid, *P. westermani*, *T. gondii*, cysticercus, and *C. sinensis*, which may be related to the dietary history of people. The Xiangjiang, Zishui, Yuanjiang, and Lishui rivers run through Hunan Province, so there are many small rivers and streams, which are conducive to the breeding of crabs, freshwater snails, freshwater fish, and shrimps. The local people have a habit of eating freshwater creatures and drinking raw water from streams, which greatly increases the risk of parasitic infection, such as *P. westermani* and *C. sinensis* (Peng et al., 2018; Li et al., 2019). A recent study found that the infection status of plerocercoid in snakes was severe in Hunan Province (Liu et al., 2020), while eating raw snake galls, using frog skin for injury and disease, and eating snakes and frogs were very common for local residents (Liu et al., 2010; Wang et al., 2011; Wang et al., 2014; Zhang et al., 2020). Hunan Province is one of the most important provinces in pork and mutton production, and wild snakes have been widely sold at food markets in some regions. Especially, foodborne parasites are usually zoonotic, so livestock usually can be infected with parasites, such as *T. gondii*, cysticercus, and *Trichinella* (Cui et al., 2013; Jiang et al., 2016; Li et al., 2016; Tan et al., 2018; Wang et al., 2019). Eating undercooked meat is popular in some areas of this province, which increases the risk of human parasitic infection with parasites.

With enhanced globalization as well as international and regional communication and cooperation, imported cases have become the main challenge to the elimination of several parasitosis, such as malaria and schistosomiasis, in mainland China (Song et al., 2018). A total of 31,740 cases of infectious diseases mainly from Africa and Asia were imported to mainland China during 2005–2016, and most of them were found in Yunnan Province (Wang et al., 2018). In this survey, we found three imported cases outside of Hunan Province, two of which were from Africa, and the imported *S. mansoni* myelopathy was the first case reported in China. The frequency of imported infection is increasing in China, and transmission of infection through international travel arises this health issue. Between January 1, 2014, and December 31, 2016, 22,797 cases were identified among 805,993,392 travelers arriving in China, with an incidence of 28.3 per million (Fang et al., 2018). In recent years, Kala-azar has been endemic and persistent in the mid-west regions of China. The reported cases were mainly from Xinjiang, Gansu, and Sichuan (Zheng et al., 2017). In general, we still need to step up efforts to prevent and control imported diseases.

In China, the relevant examinations in the parasitology diagnostic laboratory mainly include etiological and immunological assays. The examination of etiology is mainly used to observe whether there are worms, eggs, or trophozoite of protozoa in specimens through a microscope, but the detection rate of this method is very low (Lin et al., 2011). In recent decades, the immunological technology in the medical industry has been more and more mature, with a higher sensitivity and positive rate than that of etiological examination, and the detection of parasitosis is also more and more inseparable from immunological examination (Pacheco et al., 2020). That is why sometimes the positive result by immunological examination is negative by etiological examination. The diagnosis of parasitic diseases mainly depends on clinical manifestations and immunological examination, but immunological examination is only an auxiliary diagnosis method, which cannot be used as the basis for the diagnosis of parasitosis. The positive result of etiological examination is the direct evidence to confirm parasitic infection. There are still many limitations of current detection methods. On the one hand, both microscopy and immunological assays are labor-intensive and time-consuming, have low sensitivity and low specificity, and require professionals to clarify the results (McHardy et al., 2014). On the other hand, factors influencing the sensitivity of parasitic examinations include sporadic shedding (requiring the examination of multiple feces specimens), patient medications, sample collection interval, and the preservation of specimens prior to testing. Additionally, false positives in immunodetection may be related to the wrong time of reading results, improper dilution of specimen, use of out-of-date methods, and improper storage temperature of samples (Roellig et al., 2017). Considering the limitation of microscopic and immunological tests, molecular tests such as DNA-based and quantitative PCR (qPCR)-based detection methods have been gradually developed for parasite detection. Although molecular assays are more rapid and sensitive, the cost of many of them is much higher than traditional ones. Thus, a molecular test has not been extensively used in commercial laboratories (Verweij and Stensvold, 2014; van Lieshout and Roestenberg, 2015; Paulos et al., 2016; Ryan et al., 2017).

We observed an age distribution pattern among the patients, and the positive rate of people under the age of 15 was relatively high especially for foodborne parasites (**Figure 3**). Some children and teenagers at this age live with their grandparents, and they spend lots of time playing outside without adult supervision such as playing in the river and eating undercooked freshwater fish, shrimps, and crabs, which would increase the risk of parasitic infections. The largest number of people tested for parasitic disease was between 50 and 69; most middle-aged people at this age are farmers, living in the village; and they usually work barefoot in the fields or in rivers. Some of them prefer drinking raw water and eating raw fish, shrimp, crab, and snake galls, and they work in areas where parasitic diseases are prevalent, which greatly increases the risk of parasitic infections.

Located in the middle reaches of the Yangtze River and the south of Dongting Lake, Hunan Province is surrounded by mountains and hills to the east, south, and west, while the central and northern parts are a U-shaped basin, open in the north. The Xiangjiang, Zishui, Yuanjiang, and Lishui rivers converge on the Yangtze River at the Dongting Lake. It has a complex terrain and a subtropical monsoon humid climate with a mild climate, four distinct seasons, and concentrated rainfall. Warm and humid climates are ideal for parasites to grow and reproduce, so parasitosis has long been a major public health problem in Hunan Province. The prevalence of overall parasitic infection in different regions of Hunan Province ranged from 6.49% to 22.10%, with the highest and lowest prevalence in Yongzhou and Xiangtan, respectively (**Figure 4**). The difference of prevalence of several common parasites in different regions was also significant (*P* < 0.05), except for cysticercosis. The number of species was bigger, and the intensity of parasitic infection was greater in people who live in mountainous areas than those in hilly areas. People who lived in lake areas had the lowest parasitic infection, but *S. japonicum* infection was much more serious in lake areas and hilly areas. For example, Yueyang, Yiyang, and Changde are close to Dongting Lake, Wanzi Lake, and Dalian Lake, respectively, where *Oncomelania hupensis*, the intermediate host of *S. japonicum*, are abundant. That is why we found a relatively high positive rate of *S. japonicum* in these cities. This discovery was consistent with that found in goats in Hunan Province (Ma et al., 2014). There is a higher intensity and longer duration of precipitation in mountainous areas than that of hilly areas, which is suitable for parasites to multiply. In addition, more goats are raised in mountainous areas where people eat mutton more easily and frequently. Some people even prefer to eat undercooked lamb which increases the risk of parasitic infections. There are a lot of wild animals in mountainous areas, most of which are infected with parasites. An investigation in 2018 found that the prevalence rate of plerocercoid infection in wild snakes from Hunan Province was up to 91.7% (Liu et al., 2020). Eating raw snake galls and snakes was very common for the local people because many people believe snake galls possess therapeutic effects on many diseases and snake meat is a great health tonic. Such customs in this province may facilitate human infection with plerocercoid. Fortunately, China has now banned the sale of wild animals in the market, which may greatly reduce the risk of foodborne parasite infection in the future.

The prevalence of some parasites and their intermediate hosts is affected by season, climate, temperature, etcetera. For example, the snail is the intermediate host of *S. japonicum*, and there are more snails in spring and autumn, so the prevalence of *S. japonicum* is relatively high in the two seasons. In this study, there was a significant difference in the prevalence of parasitosis between months, which is consistent with a previous study (Wang et al., 2019). Additionally, a survey of helminths in goats in Hunan Province shows that the number and intensity of nematodes and cestodes peaked in summer and autumn, while the highest prevalence of trematodes was in winter (Ma et al., 2014).

In recent years, epidemic prevention and control departments have also carried out various prevention and control measures, such as changing the water supply, improving the flush system, taking medicine, and health education (De-Jian et al., 2013; Wang et al., 2016). The positive rate of soil-transmitted nematodes, such as hookworm, *T*. *trichiura*, and *A*. *lumbricoides*, was significantly reduced. Since the outbreak of COVID-19, the CPC Central Committee has attached great importance to the public health threat posed by overeating wild animals and introduced laws to strictly prohibit the predation of wild animals, which is conducive to reducing the infection of foodborne parasites. However, there is still much to do to prevent and control foodborne parasites.

## 5 Conclusions

The incidence of soilborne nematode infections has decreased significantly. However, foodborne parasites gradually become the main parasitic infections and multiple infections become more common. This study is of great significance evaluating the prevalence of parasitosis in Hunan Province in recent years, which suggests that we should not only continue the prevention and control of soilborne nematodes but also focus on the prevention and control of foodborne parasites.

### 5.1 Limitation

Though this retrospective study provided useful epidemiological information on the current parasitic infection situation in Hunan Province, which will be useful for parasitosis control, it is not devoid of limitations. First, people suspected to have a parasitic infection by clinicians or themselves were recommended to our laboratory for further testing. Therefore, the positive rate of this study may be higher than that of stratified cluster random sampling method. Second, some detailed data of cases were not recorded, such as race, occupation, and education level, so we could not conduct a multifaceted analysis. Another imperfection is that we did not follow-up the positive patients regularly so that we were unable to analyze the cure status of these patients.

## Data Availability Statement

The raw data supporting the conclusions of this article will be made available by the authors, without undue reservation.

## Ethics Statement

The studies involving human participants were reviewed and approved by the Ethics Committee of the School of Basic Medical Science, Central South University, Changsha, China. Written informed consent to participate in this study was provided by the legal guardian/next of kin of the participants.

## Author Contributions

XL: conceptualization, provision of photographs, laboratory analyses, data analysis, writing—original draft preparation, writing—review and editing, and project administration. MW: laboratory analyses and raw data statistics. YL: laboratory analyses and review manuscript. JL: laboratory analyses. DY: laboratory analyses. LJ: conceptualization, data analysis, writing—review and editing, project administration, and funding acquisition. All authors contributed to the article and approved the submitted version.

## Funding

This work was supported by the National Natural Science Foundation of China (grant number 32170510); Natural Science Foundation of Hunan Province, China (grant number 2020JJ4765); Open-End Fund for the Valuable and Precision Instruments of Central South University (grant number CSUZC2019046); Science and Technology Program of Hunan Province (grant number 2021ZK4154); and Graduate Case Base Construction Project of Central South University (grant number 2020ALK91).

## Conflict of Interest

The authors declare that the research was conducted in the absence of any commercial or financial relationships that could be construed as a potential conflict of interest.

## Publisher’s Note

All claims expressed in this article are solely those of the authors and do not necessarily represent those of their affiliated organizations, or those of the publisher, the editors and the reviewers. Any product that may be evaluated in this article, or claim that may be made by its manufacturer, is not guaranteed or endorsed by the publisher.
